# Cutaneous leishmaniasis in northwestern Saudi Arabia: identification of sand fly fauna and parasites

**DOI:** 10.1186/s13071-017-2497-6

**Published:** 2017-11-02

**Authors:** Najoua Haouas, Omar Amer, Fawwaz Freih Alshammri, Shorooq Al-Shammari, Latifa Remadi, Ibrahim Ashankyty

**Affiliations:** 1 Laboratoire de Parasitologie-Mycologie Médicale et Moléculaire (LR12ES08), Département de Biologie Clinique B, Faculté de Pharmacie, Université de Monastir, Monastir, Tunisia; 2 College of Applied Medical Sciences, Clinical Laboratory Sciences Department, University of Hail, Hail, Kingdom of Saudi Arabia; 3 Dermatology service, King Khaled Hospital, Hail, Kingdom of Saudi Arabia

**Keywords:** Cutaneous leishmaniasis, *Leishmania major*, Sand fly, Hail, Northwestern Saudi Arabia

## Abstract

**Background:**

Cutaneous leishmaniasis (CL) is a vector-borne disease transmitted by the bite of an infected sand fly. This disease is highly prevalent in Saudi Arabia where *Leishmania major* and *L. tropica* are the etiological agents. In the region of Hail, northwestern of Saudi Arabia, the incidence is about 183 cases/year. However, the epidemiology of the disease in this area is not well understood. Thus, an epidemiological survey was conducted in 2015–2016 to identify the circulating parasite and the sand fly fauna in the region of Hail. Skin lesion scrapings were collected from suspected patients with CL.

**Methods:**

The diagnosis was made by microscopic examination of Giemsa-stained smear and PCR. The parasite was identified by PCR and sequencing of the single copy putative translation initiation factor alpha subunit gene. Sand fly specimens were collected and identified morphologically. Total DNA was extracted from the abdomen of female specimens and *Leishmania* DNA was detected by PCR.

**Results:**

Among the 57 examined patients, 37 were positive for CL. The identification of the parasite has revealed the single species *Leishmania major*. The 384 sand flies were collected belonged to two genera (*Phlebotomus* and *Sergentomyia*), six sub-genera and six species. *Phlebotomus papatasi*, *Ph. kazeruni* and *Sergentomyia clydei* were the dominant species. *Leishmania* DNA was detected in two females of *Ph. papatasi* two of *Ph. kazeruni* and one specimen of *Sergentomyia clydei*.

**Conclusions:**

*Leishmania major* is confirmed to be the etiological agent of cutaneous leishmaniasis in northwestern Saudi Arabia. The molecular detection of *Leishmania* DNA in *Ph. papatasi* and *Ph. kazeruni* supports the potential role of these two species in the transmission of *Leishmania*. Further epidemiological studies are needed to prove their role and to evaluate the burden of CL in the study region.

## Background

Cutaneous leishmaniasis is a parasitic disease caused by a flagellated protozoan belonging to the genus *Leishmania*. It is known to be the ninth largest disease burden among the 13 parasitic and bacterial neglected tropical diseases worldwide [1, 2]. Although it is self-healing, CL causes skin ulcers and disfiguring scars that can result in serious social and psychological stigma [3–5]. Because of the devastating consequences to the patient, CL is recognized as a special public health problem. In the Middle East and across to central Asia, CL is endemic with an average estimated annual incidence of 321,300 cases [6]. Among countries of this region, Saudi Arabia was reported as the fourth most endemic focus of zoonotic CL after Afghanistan, Iran and Pakistan with an estimated incidence ranging from 9600 to 15,800 cases/year [6]. In this region, the first real documented cases date from 1973 [7]. Many Saudi provinces are endemic for CL including the Al-Hassa oasis, Al-Madinah Al-Munawarah and Al Qassim provinces where desert rodents (*Psammomys obesus* and *Meriones libycus*) are the main reservoir hosts and the vector *Ph. papatasi* is prevalent [8–11]. In most endemic areas in Saudi Arabia, the causative organism was identified as *L. major.* Cutaneous leishmaniasis due to *L. tropica* is less prevalent compared to zoonotic CL caused by *L. major*. It occurs within small endemic foci in the west (Al Madina Al-Munawarah and Al Qassim) and southwest (high plateau of Aseer) provinces [9, 10, 12–15].

Despite the large distribution of this parasitic disease, studies focusing on the identification of the *Leishmania* species and the sand fly fauna are rare. Indeed, the characterization of the parasite circulating in this geographical area only started at the beginning of the 1990s. Since then and up to now only about 224 *Leishmania* isolates have been identified to the species level either by isoenzymatic methods (*n* = 38 isolates) or using a molecular approach (from culture and human clinical samples *n* = 102 and sand flies *n* = 84) [9, 10, 13, 16–18].

Several sand fly investigations have described phlebotomine species composition, their geographical distribution and their role in disease dynamics in Saudi Arabia. These surveys have revealed the presence of 25 species with a predominance of *Ph. papatasi* in all investigated areas of the kingdom [19–22]. Moreover, it was demonstrated that the geographical distribution of CL coincides with the distribution of the sand fly vectors *Ph. papatasi* and *Ph. sergenti* [20, 23].

According to the Saudi Ministry of Heath seven-year reports (2006–2012), Hail Province, Northwestern Saudi Arabia, is reported as the fifth most infected region among the 20 Saudi provinces after El Qassim, Al-Madinah Al-Munawarah, El Hassa and Riyadh [24]. Indeed, the average reported incidence of CL in Hail was 183 cases/year. Despite the endemic state of Hail province to CL, no published report is available describing the epidemiological profile of this disease including the identification of the parasite and sand fly species and their geographical distribution.

Here, we aimed to identify both the *Leishmania* species and the sand fly species composition in the Hail region in northwestern Saudi Arabia including the monthly abundance of circulating phlebotomine species. Such data are used to understand the structure of this CL focus and to clarify the role of the sand fly species in the transmission of the disease and thereby to establish effective control and preventive measures against this parasitic disease.

## Methods

### Description of the study area

**Fig. 1:**
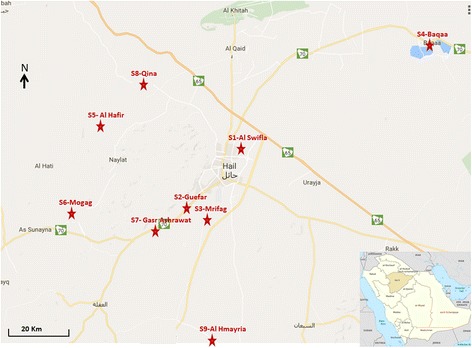
Location of sand fly collection sites (stars in red) in the Hail Province, northwestern KSA

Hail is located in the northwestern region of the Kingdom of Saudi Arabia (between 25°35′–29°00′N, 39°01′–44°45′E). It is bordered by Al Jouf to the North, Northern borders to the northeast, Al Qassim and Riyadh to the south and Tabuk and Al Madina Al Munawara to the west. Hail covers an area of almost 118,322 km^2^ and has a population of 527,000 (Ministry of the Interior, 2013 estimate) (Fig. 1). It is located at 914 m above mean sea level and has an annual rainfall of 100.6 mm. The principal part of Hail is composed of the Nafud Desert, covering about 64,000 km^2^. The weather system of Hail Region is arid to extra arid. Summer temperatures typically rise as high as 50 °C during daytime with a diurnal variation of about 25 °C. Winter temperatures hover around freezing at night especially at higher altitudes although the ground occasionally freezes and daytime temperatures nearly always reach 25 °C in the sun.

### Sample collection

Human CL cases were collected from dermatology services in different hospitals of the Hail region. For each patient, socio-demographic (gender, age, geographical residency of patients and nationality) and clinical data (number of lesions, precise location, size, clinical aspect and evolution duration of each lesion) were recorded on a pre-printed sheet. For each patient, two samples were collected with the first used to prepare two smears for Giemsa staining and microscopic examination. The second sample was stored in phosphate buffered saline (PBS) at -20 °C for further molecular studies.

**Fig. 2:**
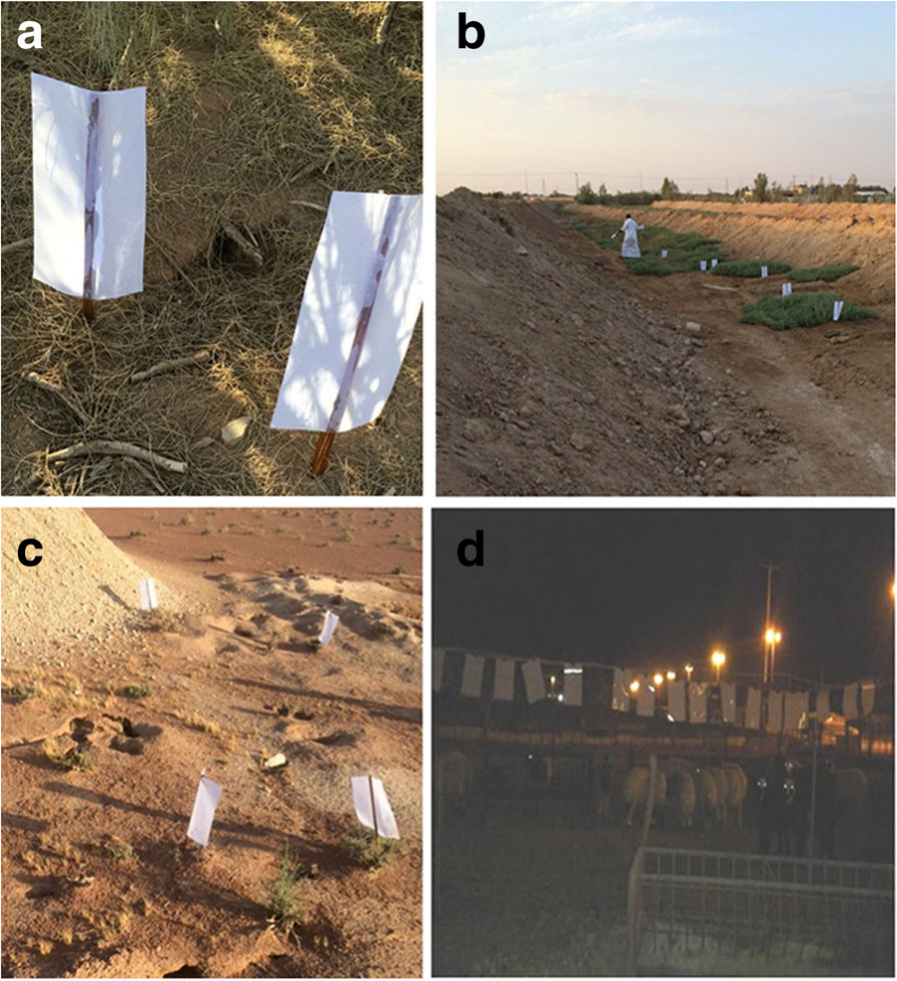
Examples of collection sites: **a** near rodent burrow; **b** in valleys; **c** sandy area; **d** inside animal stables

Sand flies were captured twice a week from September 2015 to October 2016 in nine geographical locations within Hail Province (Fig. 1) from where the regional health authorities reported human CL cases. On each occasion, thirty sticky traps (A4 white paper sheets coated with crude Castor oil) were placed in different biotopes. Indoor (animal shelters) traps were placed at 1.5 m above ground sheltered from air streams. Outdoor (at the entrance of rodent burrows and in sandy areas and valleys) traps were put on the ground with a stick as shown in Fig. 2. Traps were placed at sunset and collected the next day before sunrise.

### Molecular detection and identification of *Leishmania* parasites

DNA extraction was performed on clinical samples and female sand flies using the ReliaPrep™ gDNA Tissue Miniprep System Kit (Promega, Madison, United States), following the manufacturer’s instructions.

PCR amplification was carried out using the genus-specific primers of the conserved sequences of the small subunit ribosomal DNA (SSU-rDNA) gene (PCR-Lei) for the detection of *Leishmania* spp. infection according to the protocol of Spanakos et al. [25]. This PCR is genus-specific and does not allow the identification of the species. The forward primer Lei70L was 5′-CGC AAC CTC GGT TCG GTG TG-3′ and the reverse primer Lei70R was 5′-CGC GGT GCT GGA CAC AGG GTA-3′. PCR was performed in a final volume of 50 μl containing 1× PCR buffer, 1.5 mM MgCl_2_, 0.2 mM of each deoxynucleotide, 3 units of *Taq* DNA Polymerase (all obtained from Promega) and 100 pM of each primer. PCR was performed in a PTC 100 Thermal Cycler, with the following conditions: initial denaturation at 94 °C for 5 min, 40 cycles at 94 °C for 30 s, 65 °C for 30 s and 72 °C for 30 s and a final elongation step at 72 °C for 10 min. For each experiment, positive (DNA extracted from *Leishmania* culture) and negative (Human DNA extracted from a healthy donor) controls were used. Products were separated by 2% agarose gel electrophoresis containing 0.5 mg/ml ethidium bromide and visualized on a UV transilluminator.

Thereafter, DNA from the positive sample was re-analyzed using a second set of primers targeting a coding DNA sequence (CDS) of the putative translation initiation factor alpha subunit gene according to the protocol of El Baidouri et al. [26]. Genomic DNA was amplified by real-time PCR using SYBR Green method (Light Cycler 480 II, Roche, Rotkreuz, Switzerland). The amplified products were sequenced on both strands (Eurofins MWG Operon, Ebersberg, Germany) and the obtained sequences were blasted using Blastn algorithm against the “non-redundant” GenBank sequence database for the identification of *Leishmania* species.

### Phlebotomine sand flies identification

Sand flies were firstly washed with 90% ethanol to remove excess oil and then kept in labelled vials containing 70% ethanol. The head and posterior part of the abdomen of both male and female sand flies were dissected, cleared in Marc-Andrée solution [27] and then mounted between slide and coverslip in chloral gum medium [28]. Their abdomens and thorax were preserved in ethanol for further molecular analysis. Female and male specimens were morphologically identified by observing head and genital structures under the microscope using the morphological keys and characters of identification proposed by Croset et al. [29] and Leger et al. [30]. For each female specimen, the presence of eggs (gravid), blood (total or partial engorged) or unfed (no visible blood in the abdomen) status was recorded.

### *Leishmania* DNA detection and identification from female sand flies

After removal of the head and posterior extremity from each female specimen, the remainder of the body was transferred to individual sterilized 1.5 ml vials, from which genomic DNA was isolated using ReliaPrep™ gDNA Tissue Miniprep System kit (Promega, Madison, United States), following the manufacturer’s instructions. The maceration of the insect’s tissues was carried out with a piston pellet, and the final elution volume was 100 μl. DNA extracts were stored at -20 °C until use.

Five μl of the extracted DNA was used as a template for the PCR reaction for the detection of a *Leishmania* SSU rDNA sequence as described by Spanakos et al. [25] and detailed above. Identification of *Leishmania* species detected from female sand flies was performed by the amplification and sequencing of the putative translation initiation factor alpha subunit locus according to the protocol of El Baidouri et al. [26].

## Results

### Number of recruited human cases

From September 2015 to October 2016, 57 patients with suspected human CL were included in our study. The patients ranged from one to 65 years of age, with a median of 30 years. The sex ratio of men to women was 2.8 (men: *n* = 42; women: *n* = 15). Among them, 29 (50.87%) were Saudi and 28 (49.12%) were non Saudi (Egyptian: *n* = 10; Indian: *n* = 8; Pakistani: *n* = 4; Sudanese: *n* = 4; Bangladeshi: *n* = 1; Filipino: *n* = 1).

### Diagnosis and epidemiological characteristics of cutaneous leishmaniasis

Thirty-seven patients (64.91% of the diagnosed cases) were diagnosed as cases of CL. Among the 57 diagnosed patients, 18 (31.57%) had a positive direct examination. The diagnosis of the 39 remaining negative cases was confirmed by the detection of *Leishmania* DNA using PCR-Lei. Thus, among these 39 negative cases, 19 cases (48.71%) had shown a positive PCR result confirming their infection with the *Leishmania* parasite.

Among the 37 confirmed CL cases, 29 were males (78.37%), and 8 were females (21.62%). Their age ranged from three to 65 years old with a median age of 34 years. The majority (72.97%) of cases occurred in the age group 15–45 years. CL cases declined for the two other age groups (i.e. 8.10% in patients < 15 and 18.91% in patients > 45 years of age).

By comparing the infection rate between Saudi and non-Saudi patients, 17 (45.94%) were indigenous, and 20 (54.05%) were expatriate workers (Egyptian: *n* = 6; Indian: *n* = 6; Pakistani: *n* = 4; Sudanese: *n* = 3; and Bangladeshi: *n* = 1). All patients (Saudi and non-Saudi) confirmed that they had not left the Hail region in the previous five months before the onset of lesions. The positive cases were unequally distributed in the different regions of the Province of Hail. In fact, the majority of CL cases were from Hail city (*n* = 22, 59.45%), followed by Chinan (*n* = 7, 18.91%). Sporadic cases were from Al Rodha (*n* = 2, 5.4%), Baqaa (*n* = 2, 5.4%), Qina (*n* = 2, 5.4%), Samirah (*n* = 1, 2.7%) and Guefar (*n* = 1, 2.7%).

### Clinical features of cutaneous leishmaniasis

Among the positive CL cases, 94.6% had lesions over exposed parts of the body. The most commonly affected sites were the lower limbs (*n* = 21, 56.75%) (leg: *n* = 13; foot: *n* = 8) followed by the upper limbs (*n* = 10, 27.02%) and face (*n* = 4, 10.81%). Nevertheless, two CL lesions (5.40%) were found on the back (*n* = 1, 2.7%) and the groin (*n* = 1, 2.7%).

Among positive CL cases, the size of the lesion varied between 0.3 and 9 cm; about 40% of positive lesions were 3–5 cm (*n* = 15), whereas 27.02% were 1–3 cm. The lesions of 1 cm and > 5 cm represented 16.21% each. Among the same group, 22 patients (59.45%) had a single lesion, 10 patients (27.02%) with 2–5 lesions and 5 patients (13.5%) had 5–20.

The duration of the disease varied between three weeks to four months. Most CL cases (75.67%) presented for diagnosis one to two months after the onset of their skin lesions. However, 5.40% of the patients diagnosed their disease within three weeks and 18.91% after more than two months. The detailed data are summarized in Table 1.

**Table 1:** The clinical features of cutaneous leishmaniasis in the Hail region, the Kingdom of Saudi Arabia

Parameter	*n* (%)
Location of the lesion	
Lower limbs	21 (56.75)
Upper limbs	10 (27.02)
Face	4 (10.81)
Other	2 (5.40)
Size of the lesion (cm)	
< 1	6 (16.21)
1–3	10 (27.02)
3–5	15 (40.54)
> 5	6 (16.21)
Number of lesions	
1	22 (59.45)
2–5	10 (27.02)
5–10	3 (8.10)
> 10	2 (5.40)
Duration of the disease (months)	
< 1	2 (5.40)
1–2	28 (75.67)
2–3	4 (10.81)
> 3	3 (8.10)

An important clinical polymorphism of the CL lesions was noticed among the studied series. Among the 37 positive cases of CL, 6 different clinical forms were noted. The ulcero-crusted form was the most common (*n* = 19, 51.35%) followed by the ulcerated form (*n* = 8, 21.62%), the nodular (*n* = 4, 10.81%), the pseudotumoral (*n* = 3, 8.10%), the vegetant (*n* = 2, 5.40%) and the nodulo-papular (*n* = 1, 2.70%).

### *Leishmania* species identification in clinical samples

Among the 37 positive CL cases, 25 (including 18 positive cases by direct examination and 7 positive cases by PCR-Lei) were positive for the amplification of the single copy gene. These PCR products were sequenced and identified by comparison with the nucleotide-nucleotide Basic Local Alignment Search Tool (BLAST) (GenBank DNA sequence database, National Centre for Biotechnology Information) (www.ncbi.nlm.nih.gov/blast/). All of the 25 CL cases were identified as *L. major*. No case of *L. tropica* was identified.

### Sand fly species composition and abundance

During the entomological survey, a total of 46 weekly collections were performed from September 2015 to October 2016. In total, 384 wild-caught phlebotomine sand flies were captured including 212 males and 172 females. Among these females, four specimens were blood fed, and the abdomen of 18 others were filled with eggs. Regarding sand fly individuals, the highest number of flies were recorded in Guefar (*n* = 187), followed by Mrifag (*n* = 119) and Al Swifla (*n* = 27). However, only eight specimens were collected in Al Hmayria, seven in Mogag and a single fly in Baqaa.

The collected sand fly specimens were distributed among two genera [*Phlebotomus* (*n* = 292, 76.04%) and *Sergentomyia* (*n* = 92, 23.95%)], six sub-genera [*Phlebotomus* (*n* = 242, 63.02%), *Paraphlebotomus* (*n* = 49, 12.76%), *Larroussius* (*n* = 1, 0.26%), *Sintonius* (*n* = 81, 21.09%), *Sergentomyia* (*n* = 9, 2.34%), and *Grassomyia* (*n* = 2, 0.52%)] and six species (*Ph. papatasi*, *Ph. kazeruni*, *Ph. syriacus*, *Se. clydei*, *Se. antennata* and *Se. dreyfussi*) (Tables 2 and 3).

**Table 2:** Relative abundance and sex ratios of *Phlebotomus* spp. identified from nine collecting sites in the Hail Province

	Collection locality	Geographical coordinate	*Ph. papatasi*			*Ph. kazeruni*			*Ph. syriacus*			Total (%)		
			M (%)	F (%)	M:F	M (%)	F (%)	M:F	M (%)	F (%)	M:F	M (%)	F (%)	M:F
S1	Al Swifla	27°34′N, 41°45′E	7 (2.39)	8 (2.73)	0.875	–	1 (0.34)		–	–		7 (2.39)	9 (3.08)	0.77
S2	Guefar	27°24′N, 41°33′E	64 (21.91)	62 (21.23)	1.032	13 (4.45)	–		1 (0.34)	–		78 (26.71)	62 (21.23)	1.25
S3	Mrifag	27°22′N, 41°38′E	46 (15.75)	32 (10.95)	1.437	6 (2.05)	12 (4.10)	0.5	–	–		52 (17.80)	44 (15.06)	1.18
S4	Baqaa	27°53′N, 42°24′E	–	–		1 (0.34)	–		–	–		1 (0.34)	–	
S5	Al Hafir	27°38′N, 41°16′E	–	2 (0.68)		4 (1.36)	2 (0.68)	2	–	–		4 (1.36)	4 (1.36)	1
S6	Mogag	27°22′N, 41°10′E	1 (0.34)	2 (0.68)	0.5	4 (1.36)	–		–	–		5 (1.71)	2 (0.68)	2.5
S7	G. Ashrawat	27°20′N, 41°27′E	5 (1.71)	1 (0.34)	5	3 (1.02)	–		–	–		8 (2.73)	1 (0.34)	8
S8	Qina	27°46′N, 41°25′E	3 (1.02)	2 (0.68)	1.5	–	3 (1.02)		–	–		3 (1.02)	5 (1.71)	0.6
S9	Al Hmayria	26°55′N, 41°38′E	7 (2.39)	–		–	–		–	–		7 (2.39)	–	
	Total		133 (45.54)	109 (37.32)		31 (10.61)	18 (6.16)		1 (0.34)			165 (56.5)	127 (43.5)	1.3
			242 (82.87)			49 (16.78)			1 (0.34)			292 (100)

**Table 3:** Relative abundance and sex ratios of *Sergentomyia* spp. identified from nine collecting sites in the Hail Province

	Collection locality	Geographic coordinate	*Se. clydei*			*Se. antennata*			*Se. dreyfussi*			Total (%)		
			M (%)	F (%)	M:F	M (%)	F (%)	M:F	M (%)	F (%)	M:F	M (%)	F (%)	M:F
S1	Al Swifla	27°34′N, 41°45′E	4 (4.34)	2 (2.17)	2	2 (2.17)	3 (3.26)	0.66	–	–		6 (6.52)	5 (5.43)	1.2
S2	Guefar	27°24′N, 41°33′E	24 (26.08)	21 (22.82)	1.143	–	1 (1.08)		1 (1.08)	–		25 (27.17	22 (23.91)	1.13
S3	Mrifag	27°22′N, 41°38′E	13 (14.13)	9 (9.78)	1.444	1 (1.08)	–		–	–		14 (15.21)	9 (9.78)	1.55
S4	Baqaa	27°53′N, 42°24′E	–	–		–	–		–	–		–	–	
S5	Al Hafir	27°38′N, 41°16′E	1 (1.08)	4 (4.34)	0.25	–	–		–	–		1 (1.08)	4 (4.34)	0.25
S6	Mogag	27°22′N, 41°10′E	–	–		–	–		–	–		–	–	
S7	G. Ashrawat	27°20′N, 41°27′E	–	–		–	–		–	–		–	–	
S8	Qina	27°46′N, 41°25′E	–	2 (2.17)		–	2 (2.17)		1 (1.08)	–		1 (1.08)	4 (4.34)	0.25
S9	Al Hmayria	26°55′N, 41°38′E	–	1 (1.08)		–	–		–	–		–	1 (1.08)	
	Total		42 (45.65)	39 (42.39)		3 (3.26)	6 (6.52)		2 (2.17)			47 (51.08)	45 (48.91)	1.04
			81 (88.04)			9 (9.78)			2 (2.17)			92 (100)		

*Sergentomyia dreyfussi* was exclusively found at the station of Guefar and Qina (Table 3). Among the *Phlebotomus* species caught, *Ph. papatasi* was the most predominant (82.87%) species, followed by *Ph. kazeruni* (16.78%) and *Ph. syriacus* (0.34%). For the genus *Sergentomyia*, *Se. clydei* represents 88.04% of the collected specimens, followed by *Se. antennata* (9.78%). *Se. dreyfussi* was rare and represented just 2.17% of the collected *Sergentomyia* flies. All blood-fed female sand flies were identified as *Ph. papatasi*.

Overall, the male to female sand fly ratio was 6:5 (212/172). This sex ratio (M/F) varied according to the sand fly species and the station (Tables 2 and 3). For all of the three identified *Phlebotomus* species, the males outnumbered the females. However, in case of *Se. antennata* the ratio of male/female is 0.5, whereas *Ph. syriacus* and *Se. dreyfussi* were only represented by males.

**Fig. 3:**
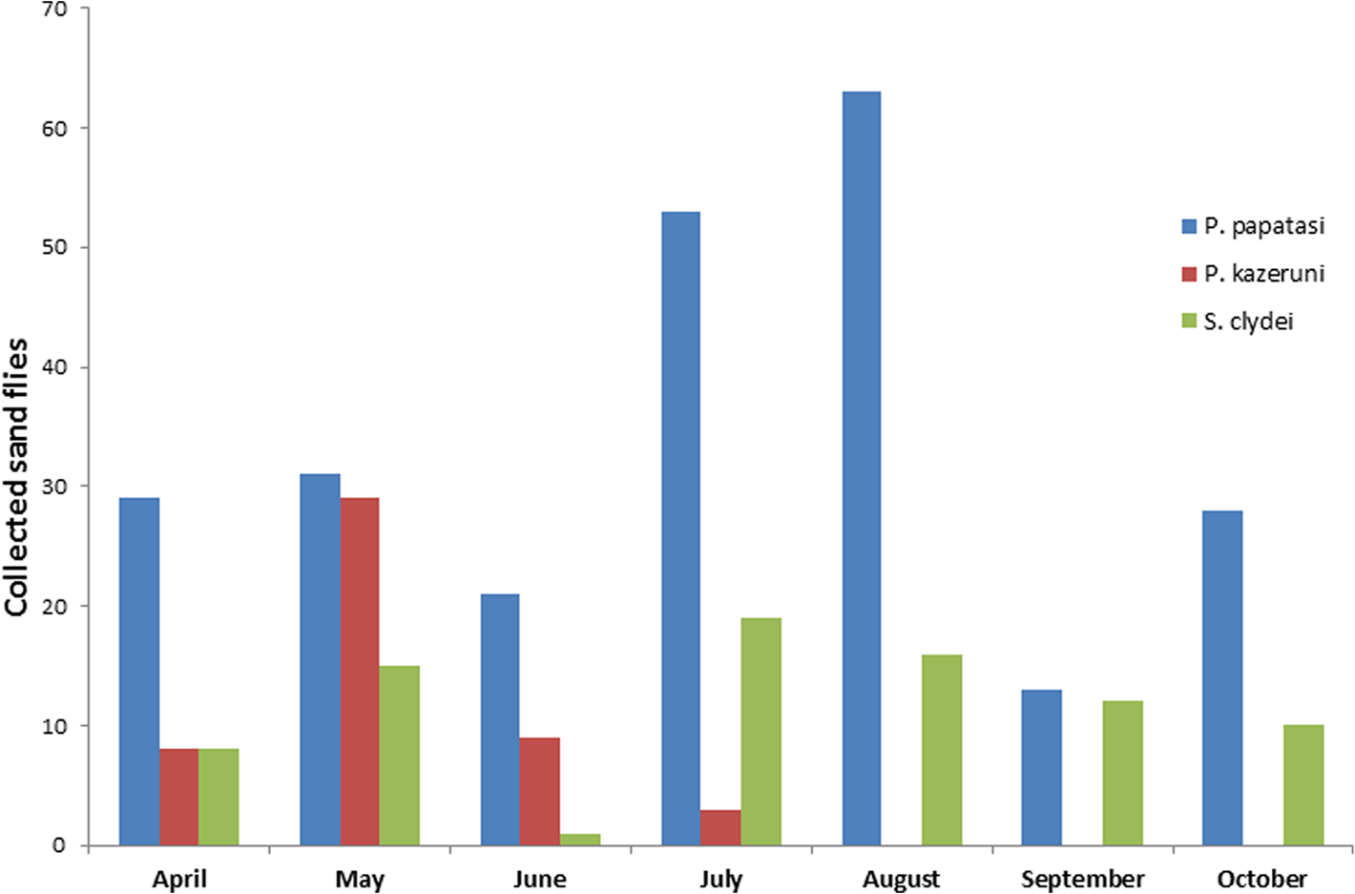
The monthly abundance of the most common sand fly species (*Ph. papatasi*, *Ph. kazeruni* and *Se. clydei*) in the Hail Province, northwestern KSA

The monthly abundance (fly/month) was examined for the three common sand fly species *Ph. papatasi*, *Ph. kazeruni* and *Se. clydei* (Fig. 3). In general *Ph. papatasi* and *Se. clydei* sand fly species were active from April to November with increased activity between July and August indicated by prominent peaks during these two months. However, *Ph. kazeruni* species had shown a peak of activity in May which markedly decreased in June and July, and the species disappeared from August when the temperature exceeded 40 °C.

### *Leishmania* DNA detection in female sand flies

Molecular detection of *Leishmania* was carried out using individual DNA extract prepared from 75 sandflies female specimens (44 *Ph. papatasi*, 15 *Ph. kazeruni*, 14 *Se. clydei* and 2 *Se. antennata*). *Leishmania* DNA was detected using SSU rDNA sequence as described by Spanakos et al. [25]. The infection rate was calculated as 6.66% (5/75). Among these five infected specimens, two were *Ph. papatasi*, two *Ph. kazeruni* and one *Se. clydei*. All of them were non-blood-fed females. The presence of *Leishmania* DNA was further confirmed by sequencing of the PCR products using the same primers as the amplification step.

The DNA extract of the five infected specimens was used for a second PCR amplifying a single copy housekeeping gene to identify the *Leishmania* species. Although the PCR was repeated on five separate occasions, no specimen was found positive for *Leishmania* DNA. This is the consequence of the low sensitivity of this second PCR compared to the first one which amplifies multi-copy DNA sequence.

## Discussion

Cutaneous leishmaniasis is endemic in Hail. According to the Ministry of Health of the Kingdom of Saudi Arabia (2006–2012) reports, about 183 CL cases are reported each year in this region [24]. Despite its endemic state, this disease is rarely studied in the Hail region. A single retrospective study was carried out in 2014 emphasising the extension of this disease in different parts of Hail [31]. No data are available concerning the causative species of this disease or the vector. For this reason, the purpose of our study was to identify the *Leishmania* species causing the disease and the sand flies species circulating in the Hail region.

To our knowledge, this is the first time that *Leishmania* species are identified in this study area. All positive human CL cases were caused by *L. major* indicating the presence of a homogeneous focus of zoonotic cutaneous leishmaniasis (ZCL) in this study area. Nevertheless, the absence of human CL cases caused by *L. tropica* in our study did not exclude the presence of small micro-foci of anthroponotic CL (ACL) in Hail region. Further studies are needed to investigate all Hail regions to affirm or deny the presence of ACL since this nosogeographical CL form is less prevalent than ZCL. This last form is endemic in many regions of Saudi Arabia including Al-Hassa Oasis, Al-Madinah Al-Munawarah and Al Qassim provinces where desert rodents (*Psammomys obesus* and *Meriones libycus*) are the main reservoir hosts [8–11, 19]. However, ACL caused by *L. tropica* was reported only in small foci such Aseer in the southwestern region [9, 10, 13, 14].

Variation in the clinical presentation of CL was noticed among the positive cases. Six different clinical forms were noticed. The ulcero-crusted form was the most common clinical form followed by the ulcerated form then the nodular form. The same result was reported in Tunisia by Masmoudi et al. [32] who noticed 54.9% of the diagnosed patients with this clinical form [32]. Also, the same study had reported the presence of nine clinical forms among a studied population of 102 cases of CL. Douba et al. [33] have reported five types of CL in the region of Aleppo, Syria. Among them, the two most common types were the papulonodular and plaque forms.

It is important to highlight that the size of the lesion is directly linked to the duration of infection. Here, small nodular lesions were noted among patients who have diagnosed their disease within one month or less since their onset. It is known that a single *Leishmania* species can elicit a range of clinical patterns [34–36]. The specific factors that influence the clinical outcome of these infections remain to be completely elucidated but likely is influenced by host genetics or immune responses [37], potentially different sand fly vector species or populations [38, 39] and the presence of *Leishmania* spp. Hybrids [40, 41]. Clinical polymorphism could also be explained by the degree of virulence of the *Leishmania* strain causing the lesion. Indeed, recent studies have reported that the degree of virulence of *Leishmania* decreases in the presence of a mutation in the gene encoding a protein BBSome complex or the gene encoding the enzyme Sphingosine kinase [42]. Further, Zangger et al. [43] showed that the virulence *of L. guyanensis* is due to its co-infection by a virus LRV (*Leishmania* RNA virus). Major efforts are still needed to understand the real causes of the observed clinical polymorphism better.

The second part of our study was an entomological survey of sand flies in the Hail Province of Saudi Arabia. The goal of this investigation was to identify the species of sand fly and assess the infection rate in female specimens. Thus, six species were identified including three of the genus *Phlebotomus* (*Ph. papatasi*, *Ph. kazeruni* and *Ph. syriacus*) and three of the genus *Sergentomyia* (*Se. antennata*, *Se. clydei* and *Se. dreyfussi*). *Phlebotomus papatasi* was the most abundant species identified in the current study. This result is consistent with the epidemiological state of leishmaniasis in Hail where ZCL is the extended noso-geographical form in this study. It is known that in Saudi Arabia, ZCL is transmitted to humans from infected rodent reservoir hosts (*Psammomys obesus* and *Meriones libycus*) through the bites of the sand fly vector *Ph. papatasi* [44]. The same species was also reported as the predominant sand fly species in many parts of the Kingdom of Saudi Arabia, such as Al-Madinah Al-Munawarah [10, 23], Jeddah [45], Riyadh [46] and Al-Qassim [47]. Since 1985, Killicki-Kendrick et al. [44] have isolated *L. major* MON-26 from a specimen of *Ph. papatasi* is proving its role as a vector of ZCL. A study on the seasonal abundance of *Ph. papatasi* in Riyadh had demonstrated that the greatest number of sand flies occurred most commonly in the summer with two peaks in June and September [46].

It is important to highlight that a complete survey carried in Saudi Arabia to study its phlebotomine sand fly fauna was that of Lewis & Buttiker [21] in 1982. During this survey, 25 phlebotomine species were identified in the whole of Saudi Arabia [21]. Inside this study area, sand fly fauna was identified in the Hail region. Between 1975 and 1980 only 14 specimens were collected in Hail, and only two species were identified: *Se. clydei* (79%) and *Se. antennata* (21%). During the same investigation, the second survey in Hail was carried in 1981, which identified *Ph. kazeruni*, *Ph. syriacus*, *Ph. naqbenius*, *Se. christophersi*, *Se. clydei*, *Se. fallax* and *Se. tiberiadis*. Since 1981, no study has been carried out in this region. Here, we identify for the first time *Se. dreyfussi* from two specimens. More entomological investigations are crucial to study the abundance of this species in the different parts of Hail.

Assessment of the *Leishmania* infection rate in sand flies is of paramount importance, and PCR-based approaches have been successfully used for detection of parasite DNA in sand flies [48–50]. The detection of *Leishmania* DNA in two unfed females of *Ph. papatasi* caught in a rural area of Hail provides additional evidence in favour of its role as a potential vector for *L. major* in this study area. Also, the detection of *Leishmania* DNA in two unfed *Ph. kazeruni* specimens (a close species to *Ph. sergenti*) suggests a potential role of *Paraphlebotomus* spp. in the transmission of *L. tropica*. It should be pointed out that the molecular detection of *Leishmania* DNA in sand flies is not enough to incriminate these insects as vector conclusively. Such finding could be explained by recent feedings on reservoirs resulting in parasite DNA remnants after blood meal digestion. Thus, isolation of the parasite from the midgut of these sand flies and its isoenzymatic identification remain mandatory to confirm this hypothesis. Finally, one specimen of *Se. clydei* was also found to be positive for the detection of *Leishmania*. In the Old World, phlebotomine species of the genus *Sergentomyia* are known as reptile-biting sand flies transmitting *Sauroleishmania* or the “lizard *Leishmania*” parasite [51]. Nevertheless, some recent investigations in Tunisia and Portugal have reported the detection of *L. major* and *L. infantum* DNA in *Se. minuta* [48, 52, 53] suggesting its potential role in the transmission of mammal-infecting *Leishmania*. Also, Senghor et al. and Berdjane-Brouk et al. suggested that *Sergentomyia* species could be involved in the transmission of *L. major* and *L. infantum* in Africa [54, 55]. To confirm this hypothesis, it will be crucial to demonstrate that *Sergentomyia* species feed on humans and can support the complete development of the parasite in natural conditions after the digestion of an infectious blood meal.

## Conclusion

In conclusion, *L. major* is the parasitic agent responsible for CL in Hail, Saudi Arabia. It exhibits a large clinical polymorphism and consequently should be included in the differential diagnosis of many common and uncommon dermatological diseases. The life-cycle of *L. major* is still not elucidated in this area but the detection of *Leishmania* DNA in two specimens of *Ph. papatasi* is evidence for the role of this sand fly species in the transmission of *L. major*. Further studies including the isolation and identification of the parasite from sand fly are needed to prove this hypothesis. Entomological data showed that *Ph. papatasi, Se. clydei* and *Ph. kazeruni* are the most dominant species in our collection. The detection for the first time of *Leishmania* DNA in two specimens of *Ph. kazeruni* supports the vector status of this species. Our investigation has provided a valuable set of data about the epidemiological features of CL in Northwestern Saudi Arabia. Nevertheless, further studies in humans, vectors and potential reservoirs are needed to determine the burden of cutaneous leishmaniasis in the whole of Hail.

## Abbreviations

CL: cutaneous leishmaniasis
ZCL: zoonotic cutaneous leishmaniasis
